# Addressing the diagnostic gap in hypertension through possible interventions and scale-up: A microsimulation study

**DOI:** 10.1371/journal.pmed.1004111

**Published:** 2022-12-06

**Authors:** Lisa Koeppel, Sabine Dittrich, Sergio Brenner Miguel, Sergio Carmona, Stefano Ongarello, Beatrice Vetter, Jennifer Elizabeth Cohn, Till Baernighausen, Pascal Geldsetzer, Claudia M. Denkinger, HPACC Consortium

**Affiliations:** 1 Division of Infectious Diseases and Tropical Medicine, Heidelberg University Hospital, Heidelberg, Germany; 2 FIND, Geneva, Switzerland; 3 Nuffield Department of Medicine, Faculty of Tropical Medicine and Global Health, University of Oxford, Oxford, United Kingdom; 4 Institute of Applied Mathematics, Heidelberg University, Heidelberg, Germany; 5 Division of Infectious Diseases, University of Pennsylvania Perelman School of Medicine, Philadelphia, Philadelphia, United States of America; 6 Heidelberg Institute of Global Health (HIGH), Medical Faculty and University Hospital, University of Heidelberg, Heidelberg, Germany; 7 Africa Health Research Institute, Somkhele, South Africa; 8 Department of Global Health and Population at the Harvard T.H. Chan School of Public Health, Boston, Massachusetts, United States of America; 9 Division of Primary Care and Population Health, Department of Medicine, Stanford University, Stanford, California, United States of America; 10 Chan Zuckerberg Biohub, San Francisco, California, United States of America; 11 German Centre for Infection Research (DZIF), partner site Heidelberg University Hospital, Heidelberg, Germany; Harvard University, Brigham and Women’s Hospital, UNITED STATES

## Abstract

**Background:**

Cardiovascular diseases (CVDs) are the leading cause of mortality globally with almost a third of all annual deaths worldwide. Low- and middle-income countries (LMICs) are disproportionately highly affected covering 80% of these deaths. For CVD, hypertension (HTN) is the leading modifiable risk factor. The comparative impact of diagnostic interventions that improve either the accuracy, the reach, or the completion of HTN screening in comparison to the current standard of care has not been estimated.

**Methods and findings:**

This microsimulation study estimated the impact on HTN-induced morbidity and mortality in LMICs for four different scenarios: (S1) lower HTN diagnostic accuracy; (S2) improved HTN diagnostic accuracy; (S3) better implementation strategies to reach more persons with existing tools; and, lastly, (S4) the wider use of easy-to-use tools, such as validated, automated digital blood pressure measurement devices to enhance screening completion, in comparison to the current standard of care (S0). Our hypothetical population was parametrized using nationally representative, individual-level HPACC data and the global burden of disease data. The prevalence of HTN in the population was 31% out of which 60% remained undiagnosed. We investigated how the alteration of a yearly blood pressure screening event impacts morbidity and mortality in the population over a period of 10 years.

The study showed that while improving test accuracy avoids 0.6% of HTN-induced deaths over 10 years (13,856,507 [9,382,742; 17,395,833]), almost 40 million (39,650,363 [31,34,233, 49,298,921], i.e., 12.7% [9.9, 15.8]) of the HTN-induced deaths could be prevented by increasing coverage and completion of a screening event in the same time frame. Doubling the coverage only would still prevent 3,304,212 million ([2,274,664; 4,164,180], 12.1% [8.3, 15.2]) CVD events 10 years after the rollout of the program.

Our study is limited by the scarce data available on HTN and CVD from LMICs. We had to pool some parameters across stratification groups, and additional information, such as dietary habits, lifestyle choice, or the blood pressure evolution, could not be considered. Nevertheless, the microsimulation enabled us to include substantial heterogeneity and stochasticity toward the different income groups and personal CVD risk scores in the model.

**Conclusions:**

While it is important to consider investing in newer diagnostics for blood pressure testing to continuously improve ease of use and accuracy, more emphasis should be placed on screening completion.

## Abstract 2

In a micro-simulation study, Lisa Koeppel and co-authors explore the comparative impact of interventions for addressing the diagnostic gap in hypertension screening in low- and middle-income countries.

## Author summary

**Why was this study done?:**

- Cardiovascular diseases (CVDs) are the leading cause of mortality globally, affecting low- and middle-income countries (LMICs) disproportionally highly.
- Hypertension (HTN) is the leading modifiable risk factor for CVDs.
- The diagnosis of HTN and thus the access to treatment is hampered by the necessity of at least one repeated measurement for a final diagnosis and the operator-dependent variability of blood pressure measurement.
- It is unclear which strategies would be the most impactful to close the diagnostic gap: more accurate, easy-to-use and/or more scalable tools or better implementation strategies to reach more persons with existing tools.

**What did the researchers do and find?:**

- We developed a stochastic microsimulation model that examines the impact of possible diagnostic interventions and implementation strategies on HTN-induced morbidity and mortality in LMICs.
- The different scenarios were applied over a period of 10 years and affected the individual risk of experiencing a CVD event.
- While improving test accuracy avoids only 0.6% of HTN-induced deaths over 10 years, scaling up test coverage and completion can avoid almost 40 million HTN-induced CVD events and 14 million (13.7%) related deaths.

**What do these findings mean?:**

- This simulation demonstrates the importance of increasing the coverage of testing for HTN and the improvement of screening completion over diagnostic accuracy of HTN testing.
- Strategies to narrow the diagnostic gap in HTN should put more emphasis on screening completion.

## Introduction

Cardiovascular diseases (CVDs) are the leading cause of mortality globally; an estimated 17.9 million people die each year, which makes up about 32% of all annual deaths worldwide [1]. Low- and middle-income countries (LMICs) account for approximately 80% of these deaths. With a prevalence of about 31% globally, hypertension (HTN) is the leading modifiable risk factor for CVD and premature death worldwide [2–5]. The age-standardized prevalence of HTN increased by 7.7% in LMICs between 2000 and 2010 [6]. HTN is diagnosed if the measurement of the systolic blood pressure is above 140 mm Hg or the diastolic blood pressure is above 90 mm Hg on two different days [7]. However, the necessity of at least a repeated measurement for a final diagnosis constitutes a barrier in access to treatment and management of the condition. Moreover, the accuracy of devices for taking a blood pressure measurement can be highly operator and device dependent. Manually operated mercury or aneroid blood pressure devices are still widely used in many LMICs, relying on skilled operators and a suitable environment to correctly operate the devices and obtain an accurate reading. Automated, digital blood pressure devices can be used more easily by less trained operators in a range of environments and have the potential to obtain more accurate readings [8].

These limitations contribute to the diagnostic gap (approximately 61%), denoting the percentage of people with HTN not having completed their second measurement and being diagnosed [9]. To improve HTN diagnosis, better implementation strategies to reach more persons with existing tools or the use of more accurate, easy-to-use tools are necessary.

This simulation study aims to estimate impact on morbidity and mortality from HTN through altering the diagnostic accuracy of HTN tools or increasing the coverage of screening for hypertension to achieve more active outreach and follow-up compared to the current standard of care.

## Methodology

### Simulation study design

This study is reported as per the “Strengthening the reporting of empirical simulation studies (STRESS)” (S1 STRESS Checklist) [10].

**Fig 1:**
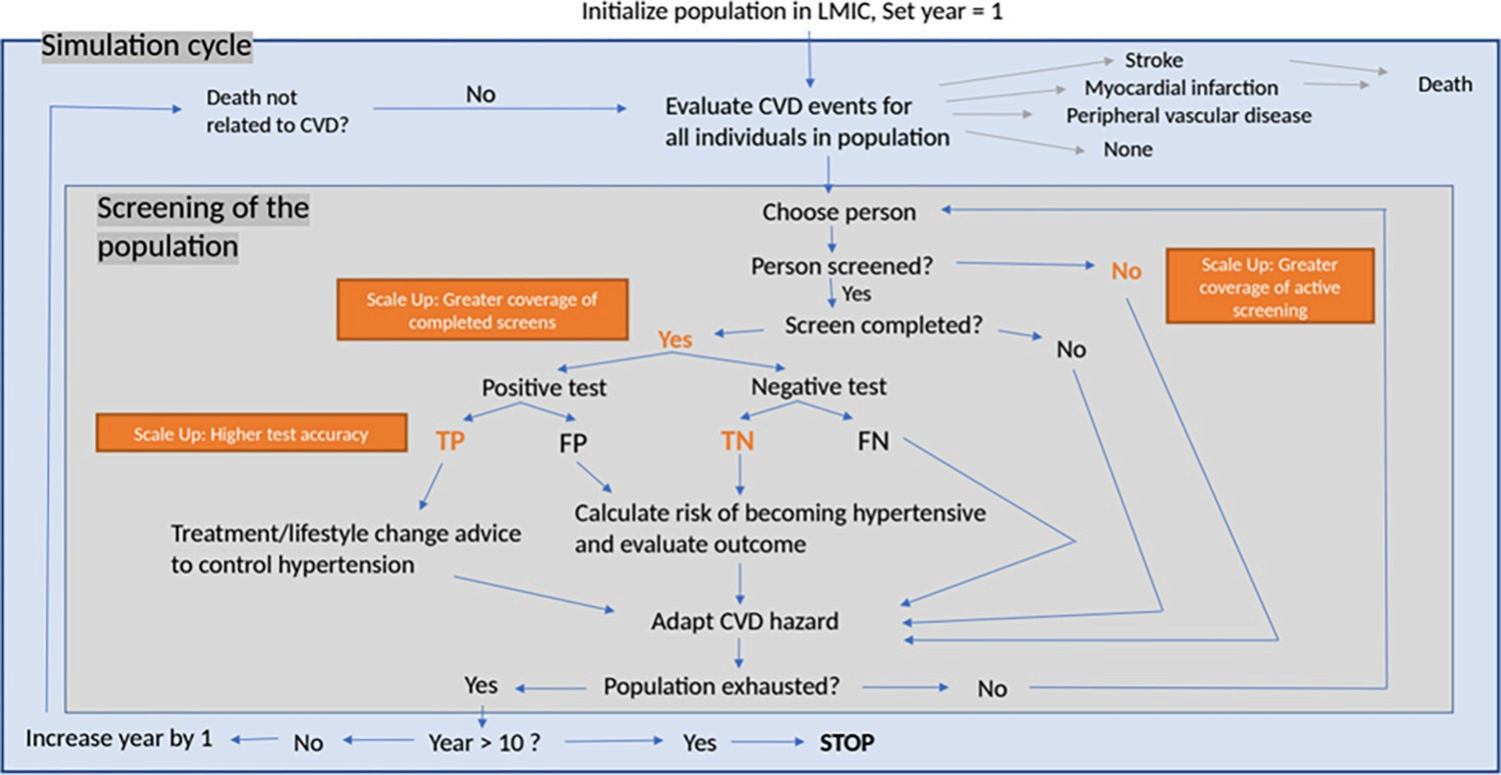
Flowchart of the microsimulation for hypertension screening scale-up. CVD, cardiovascular disease; FN, false negative; FP, false positive; LMIC, low- and middle-income country; TN, true negative; TP, true positive.

In this simulation study, we followed an artificial population representative for LMICs with respect to incidence of HTN and CVD over a period of 10 years. Every person has individual characteristics, e.g., sex or blood pressure; thus, using a microsimulation model, we were able to track each individual’s health status independently. We investigated the effect of blood pressure screening on the total number of CVD events and the resulting deaths within the population. We considered different scenarios that assumed improved accuracy or improved coverage and/or completion of screening for HTN, which is two consecutive blood pressure measurements, and compared them to the status quo. We describe the flow through the model in Fig 1.

### Population initialization

In this microsimulation, we aimed at simulating an artificial LMIC population aged 30 years and older, accounting for the fact that HTN is less prevalent in the younger generation.

Stratified by gross national income class, this would result in a population size of 3.15 billion (low-income countries (LICs): 167,113,750; lower-middle-income countries (LoMICs): 1,360,371,000; upper-middle-income countries (UMICs): 1,621,405,000). See Section A in S1 Text for further details. In order to deal with the computational complexity, we performed the calculations on respective subsets of 1 million individuals per gross national income class and scaled the outcomes to the fit the desired population size.

The characteristics of the individuals composing the LMICs population were sampled from a cross-sectional study of nationally representative, individual-level data in LMICs [11], in the following referred to “HPACC” dataset. This dataset contains the WHO STEPwise approach to noncommunicable diseases Surveillance (STEPS) data [12] and further extends it by additional population health surveys. For this analysis, a subset of solely publicly available data was used. It allowed each individual in our model to be characterized in terms of age, body mass index (BMI), sex, and HTN status and maintained correlations between the characteristics with respect to each income class. We demonstrate this by showing proportions and characteristics of the sampled subset and comparing them to the full dataset (Table A in S1 Text).

We further assigned each individual a systolic blood pressure, as the systolic blood pressure was shown to be the better determinant of CVD risk than the diastolic blood pressure [13,14]. The values were selected according to average blood pressure measurements for healthy, hypertensive, and hypertensive but treated persons and are sex and age specific (Table 1) [15]. White-coat and masked HTN are subject to the first blood pressure measurement. We only consider the final confirmatory diagnostic outcome and thus assume that this outcome reflects the true blood pressure of a person regardless of white-coat and masked HTN (Section F in S1 Text).

**Table 1:** Blood pressure distribution according to [11].

	Normal systolic blood pressure (mm Hg)			Hypertensive systolic blood pressure (mm Hg)			Treated systolic blood pressure (mm Hg)		
Age	30–39 years	40–59 years	>59 years	30–39 years	40–59 years	> 59 years	30–39 years	40–59 years	>59 years
Men	120 [114,127]	121 [114,128]	122 [116,131]	141 [133,147]	149 [139,157]	156 [143,164]	141 [129,150]	149 [134,162]	151 [135,165]
Women	114 [107,121]	117 [110,125]	122 [115,131]	138 [129,145]	148 [137,156]	157 [144,166]	133 [119,145]	146 [130,160]	150 [134,165]

Every individual was also assigned a date of death independent of CVD events. These time points were sampled by means of a Weibull distribution with mean set to the life expectancy in the respective income group (LIC: 63.5 years, LoMIC: 68.4 years, UMIC: 75.3 years; [16]) and a probability of survival adapted to the age pyramids of the representative countries (further details are described in the Section B in S1 Text) [17].

### CVD risk and death probability calculations

The mathematical risk function for experiencing a CVD event was calculated from a sex-specific Cox proportional-hazard regression (see Section C in S1 Text for further details). We adapted the calculation of the probability for a person to experience a CVD event from the Framingham heart study [18]. The Framingham risk score is based on the US population with different characteristics than individuals in LMICs, which is why we further scaled the risk by a factor accounting for the proportional CVD incidence differences in the USA and LMICs. The type of CVD event (stroke, myocardial infarction, or other CVD) and whether it led to death was determined by the relative proportion of their occurrence and the associated mortality risk (Table 2).

In case of a CVD event, we assumed a risk of death for each individual based on the CVD event that occurred. In case of survival, we evaluated the occurrence of a new CVD event by extending the CVD risk with an additional hazard for a recurrent CVD event [19–21]. To not confound the risk of occurrence and death attributed to a particular CVD event, the probability of experiencing a specific CVD was weighted by a multiplicative factor (hazard ratio) from the literature with respect to the type of patient history (Table 2). A detailed description of the risk and mortality calculation, as well as its interpolation for the remaining years, can be found in the Sections B and C in S1 Text.

**Table 2:** Parameters for the distribution of CVD events, their associated risks, and effect size of treatment used in the simulation.

*Parameter*	*Risk*									*Source*
Distribution of specific CVD event among CVDs	Myocardial infarction 46.7%, Stroke 35.5%, other CVD 17.8%									[22]
	LIC			LoMIC			UMIC			[23]
Age	30–49	50–69	>69	30–49	50–69	>69	30–49	50–69	>69	
MI mortality in first year (%)	45.2	25.7	38.4	73.6	74.3	34.9	45.2	25.7	38.4	
Stroke mortality in first year (%)	71.2	33.5	53.3	25.2	31.2	10.3	71.2	33.5	53.3	
CVD risk rescale factor to LMIC	1.100	0.053	0.302	0.837	0.558	3.134	1.100	0.053	0.302	
Risk of death from a stroke	Cumulative risk at 5 years: 60%, Cumulative risk at 10 years: 76%									[24]
Risk of death from myocardial infarction	Cumulative risk after 1 year: 21% Cumulative risk after 5 years: 41%									[25,26]
Hazard ratio for recurrent CVD	year 1: 2.4, year 3: 2.2, year 5: 2.1									[20]
Hazard ratio for recurrence of stroke	After 1 year: 15, After 5 years: 9									[21]
Hazard ratio for recurrence of myocardial infarction	1–3 years: 2.92, 3–5 years: 2.70									[19]
Risk reduction due to treatment adherence	0.80 for every reduction of 10 mm Hg in systolic blood pressure									[27]

CVD, cardiovascular disease; LIC, low-income country; LMIC, low- and middle-income country; LoMIC, lower-middle-income country; UMIC, upper-middle-income country.

### Simulation cycle

In every simulation cycle (Fig 1), we analyzed every individual’s probability for a CVD event to occur. If a CVD event was experienced, the type of CVD event (stroke, myocardial infarction, or other CVD) and whether it led to death was determined by the relative proportion of their occurrence and the associated mortality risk (Table 2 and Section C in S1 Text).

We model one screening event per year consisting of two blood pressure measurements, which is a first and a second (confirmatory) blood pressure measurement for final diagnosis. Each year the proportion of the coverage was selected randomly from the population to undergo a first blood pressure measurement. From this subgroup, a proportion was enabled to complete the screening event by a second blood pressure measurement and get diagnosed with probability depending on the different screening scenarios modeled (see below). Individuals with a higher CVD risk were more likely to complete their screening event (Section D in S1 Text). According to the outcome of the test, the risk of experiencing a CVD event was adapted. In case of a true positive test result, we quantified the impact of full dose antihypertensive treatment on the estimated CVD risk according to a meta-analysis [27]. While this is a simplification of the care cascade, we chose to make this assumption as we were primarily interested in the impact of improvements in diagnosis on the endpoints. For treated patients, we adapted the risk function by adjusting the assumed blood pressure category (Table 1).

At the end of every year, a healthy person was subject to a risk of becoming hypertensive, i.e., the blood pressure increased from normal to hypertensive (Table 1). The probability for this was predicted by means of a logistic regression model taking into account age, BMI, and sex as predictors using the HPACC dataset. See Section E in S1 Text for details. The CVD hazard was adapted based on increasing age considering the outcome of a screen or no screen, respectively. This then led to a new evaluation of the CVD events in the beginning of the following year. The last step of the simulation cycle was to determine if a person died due to reasons besides CVD events, as predefined by the life expectancy for the country income classes.

We allowed a 3-year burn-in phase for the model to create stable model outputs in the base case scenario (S0) and then performed the simulation cycle 10 times for the specific scenarios (S0 to S4). A person may experience at most one CVD event within a year. At the end of the simulation period, we investigate endpoints to compare the impact of different screening scenarios over the period of 10 years (Table 3). The results of each simulation are subject to randomness. Thus, we performed Monte Carlo simulation with 100 simulations for each income group and each scenario separately in order to obtain credible intervals for the estimators of the endpoints.

### Screening scenarios

#### (S0) Base case

We assumed that the sensitivity and the specificity of the HTN blood pressure measurement was 87% and 85%, respectively, for the baseline case [28]. The coverage, meaning how many people had at least one blood pressure measurement per year, was calculated in the following way: Geldsetzer and colleagues found that 73.6% of the population with HTN had ever had their blood pressure measured in their lifetime [9]. Adapting this number to the whole population would be an overestimate with respect to the frequency and target population, as people with HTN are more likely to complete their screening event than the population at large. Moreover, the blood pressure measurement might have taken place after many years of hypertensive status. Therefore, we considered the life years of opportunity to have blood pressure measured and scaled this accordingly. The mean age in the population was 48; thus, on average, people had 18 years opportunity to have had a measurement. Hence, the probability for undergoing one blood pressure measurement per annum reduces to 73.6%/18 = 4.09%.

The percentage of the population not having received a diagnosis is denoted as diagnostic gap and was estimated by Geldsetzter and colleagues to be 60.8% in the population with HTN [9]. Thus, 39.2% of those diagnosed with HTN completed their second screen. Analogously to above, these patients might have had a high blood pressure for years before they were diagnosed. When applying it to the whole population, we thus assumed this percentage per annum to be lower and estimated it similarly to above, that is 39.2%/18 = 2.18%. Taking into account the coverage and the percentage of two measurements, we inferred that 2.18%/4.09% * 100 = 53.26% of them completed their screen.

#### (S1) Lower accuracy of the screening event

This scenario was supposed to model the current performance of the HTN screening event under routine implementation conditions. We chose the lower bound of the confidence interval of the blood pressure measurement accuracy (sensitivity 79%, specificity 64%; [28]) represented lower expertise in the measurement. Apart from the accuracy, all other parameters were kept equal to the base case.

#### (S2) Increased accuracy of the screening event

For increased accuracy of the screening event, we chose a sensitivity and specificity of 93% and 95%, respectively. This is likely to require wider use of automated, digital devices or improved measurement techniques, although this increased accuracy is in line with the upper bound of the confidence interval of the blood pressure measurement accuracy from Irving and colleagues [28]. The remaining parameters were kept equal to the base case.

#### (S3) Increased coverage

The increased coverage represents a scale-up in the number of people screened once. This scenario assumed using already existing tests with their accuracy as outlined in the base case and would be accomplished through more accessible tools as well as programmatic improvements that translate into better implementation strategies. In this simulation, we assumed a doubling of the coverage (8.18% versus 4.09% per year in the base case), yet the percentage of having completed the confirmatory blood pressure measurement remained the same as in the base case.

#### (S4) Increased coverage + improved completion of screening event

Analogously to S3, we doubled the coverage to enable more people access to a first screen. On top of that, we modeled an improvement in the number of screening events completed. In the best case, all persons with a positive test result in the first screen will have completed their screen. This will require more accessible tools or innovation in programmatic scale-up to ensure complete follow-up.

**Table 3:** Summary of different simulation scenarios considered.

Parameter	S0 Base case	S1 Lower accuracy	S2 Improved accuracy	S3 Increased coverage	S4 Increased coverage + screens completed
Test sensitivity	87%	**79%**	**93%**	87%	87%
Test specificity	85%	**64%**	**95%**	85%	85%
% First screen per year (out of total)	4.09%	4.09%	4.09%	**8.18%**	**8.18%**
% Screen completed per year (out of population with first screen)	53.26%	53.26%	53.26%	53.26%	**90%**
% with two measurements per year (out of total)	2.18%	2.18%	2.18%	4.36%	7.11%

The bold writing indicates the variation to the base case. The percentage of persons with two measurements was inferred by the proportion of completed screens of the population with one screen.

## Results

We obtained a population size of over 3 billion (3,148,889,750) individuals comprising about 5% of individuals from LICs, 43% from LoMICs, and more than half (51.5%) from UMICs (Table 4), which is representative of the global population aged 30 and older distribution according to income. At initiation, the prevalence of HTN is lowest in LoMIC, followed by LICs, and, finally, UMICs with about one in three adults suffering from high blood pressure in the combined dataset. In total, with more than 3 billion individuals, almost 1 billion have HTN, but only 39.4% of them are aware of their diagnosis and have it treated. As such, our population largely mirrors the HPACC dataset (Table A in S1 Text) and is in line with the literature [29].

**Table 4:** Characteristics of the simulated population at initiation.

	LIC	LoMIC	UMIC	Total dataset
**Population size n (%)**	167,113,750 (5.3)	1,360,371,000 (43.2)	1,621,405,000 (51.5)	3,148,889,750
**Sex (male) n (%)**	69,128,274 (41.4)	284,434,531 (20.9)	671,864,833 (41.4)	1,025,427,638 (32.6)
**HTN n (%)**	51,462,679 (30.8)	328,584,011 (24.2)	609,859,063 (37.6)	989,905,753 (30.9)
**HTN diagnosed n (%)**	20,232,462 (39.3)	130,133,090 (39.1)	239,465,304 (39.4)	389,830,856 (39.4)
**Median BP (mmHg) [IQR]**	121 [112, 131]	119 [110, 128]	123 [113, 134]	121 [112, 131]
**Median time of death (age) [IQR]**	65 [60, 68]	70 [60, 80]	79 [69, 90]	74 [65,84]
**Overweight** **(BMI >25) n (%)**	49,625,765 (29.7)	463,614,437 (34.1)	1,112,971,306 (68.6)	1,626,211,508 (51.6)
**Median BMI [IQR]**	22 [20, 25]	23 [20, 26]	27 [24, 31]	25 [22,29]
**Median age [IQR]**	42 [35, 51]	40 [34, 45]	47 [39, 58]	42 [36,51]
**Average life expectancy (years)**	63.5	68.4	75.3	71.7

BMI, body mass index; BP, blood pressure; HTN, persons with hypertension; IQR, interquartile range; LIC, low-income countries; LoMIC, lower-middle-income countries; UMIC, upper-middle-income countries.

**Fig 2:**
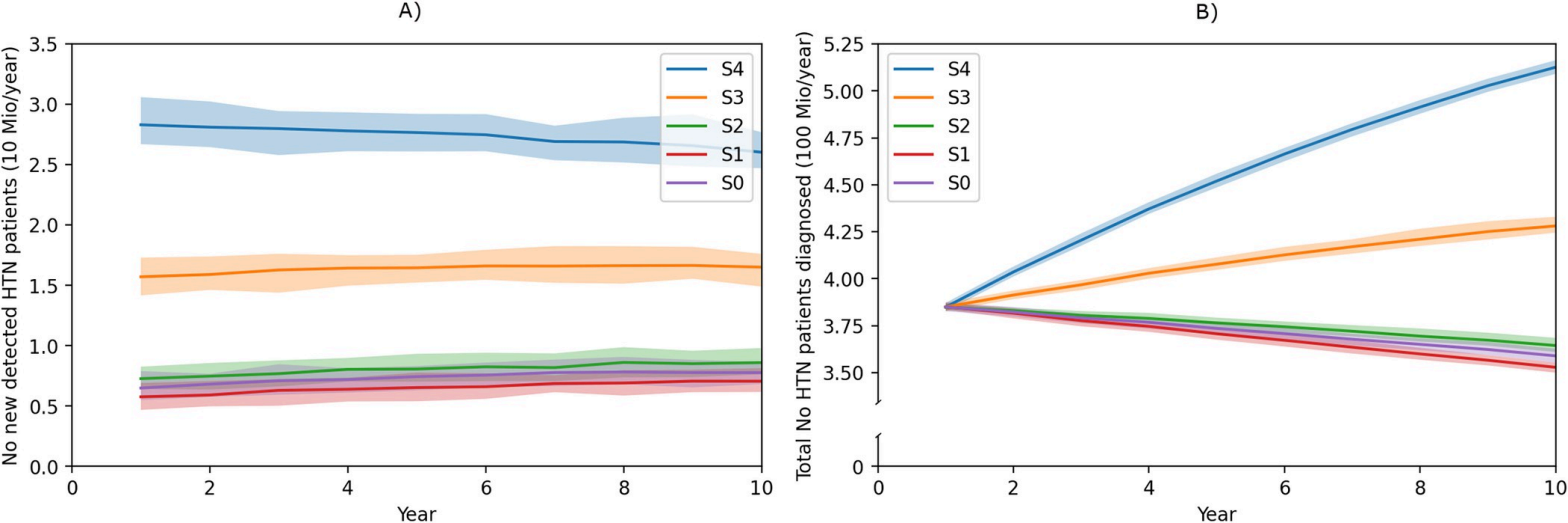
(**A**) Absolute numbers of new detected persons with HTN and (**B**) total number of detected persons with HTN for the different scenarios in LMICs; S0: Base case, S1: Lower accuracy, S2: Improved accuracy, S3: Increased coverage, S4: Increased coverage and screens completed. The bold line represents the mean value and the band the 95% credible interval. The years of model adaptation are excluded in the figure. HTN, hypertension; LMIC, low- and middle-income country.

In Fig 2, we compare the different scenarios with respect to the people diagnosed. The number of newly detected people with HTN per year is highest under scenario S4 (Increased coverage and screens completed), followed by S3 (Increased coverage) and about equal for the base case S0 and the alteration of the test accuracy S1 and S2. Only the scenarios with increasing coverage (S3+S4) are able to address the high number of undiagnosed cases of HTN. In contrast, for the baseline (S0) and the scenario where the test is more accurate but the same coverage is maintained (S2), the undiscovered burden actually further increases. This suggests that a sole increase in diagnostic accuracy is insufficient to counterbalance the rising tide of hypertension in the coming years.

**Fig 3:**
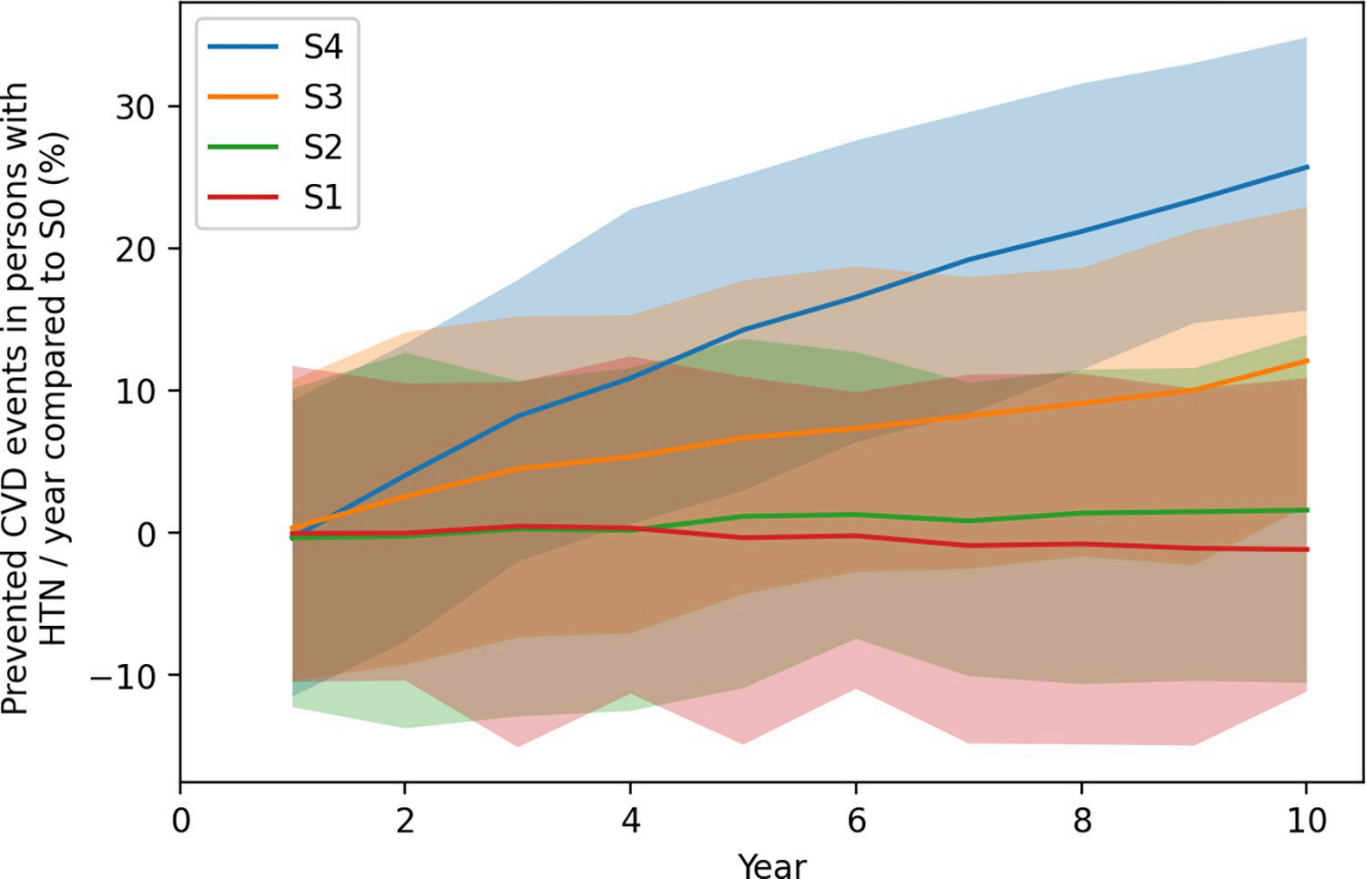
Mean percentage and 95% credible intervals of the reduction of CVD events attributable to HTN per year of the scenarios S1: Lower accuracy, S2: Improved accuracy, S3: Increased coverage, and S4: Increased coverage and screens completed compared to the baseline case S0. The bold line represents the mean value and the band the 95% credible interval. The first years of model adaptation are excluded in the figure. CVD, cardiovascular disease; HTN, hypertension.

The same findings are reflected when assessing the differences of the scenarios for the number of CVD events in the population with hypertension per year. Fig 3 displays that under scenario S4, the increased coverage and completion of a screening event has the strongest impact on preventing CVD events per year in persons with HTN. In fact, it leads to a reduction of over 7 million (7,031,120; 26%) CVD events in year 10 compared to current standard of care (scenario S0). In sum, this equals 39,650,363 (14.2%) prevented CVD events over the period of 10 years. With only increased coverage, we observe 3,304,212 prevented CVD events (12.1%) by following scenario S3 10 years after a rollout of the program. It also becomes apparent that a lower test accuracy will not be sufficient to counterbalance the expected increased burden of HTN-induced CVD events as the percentage becomes negative (−1.2) at year 10, denoting an increase in CVD events. Heatmaps are provided in Fig A in S1 Text.

**Fig 4:**
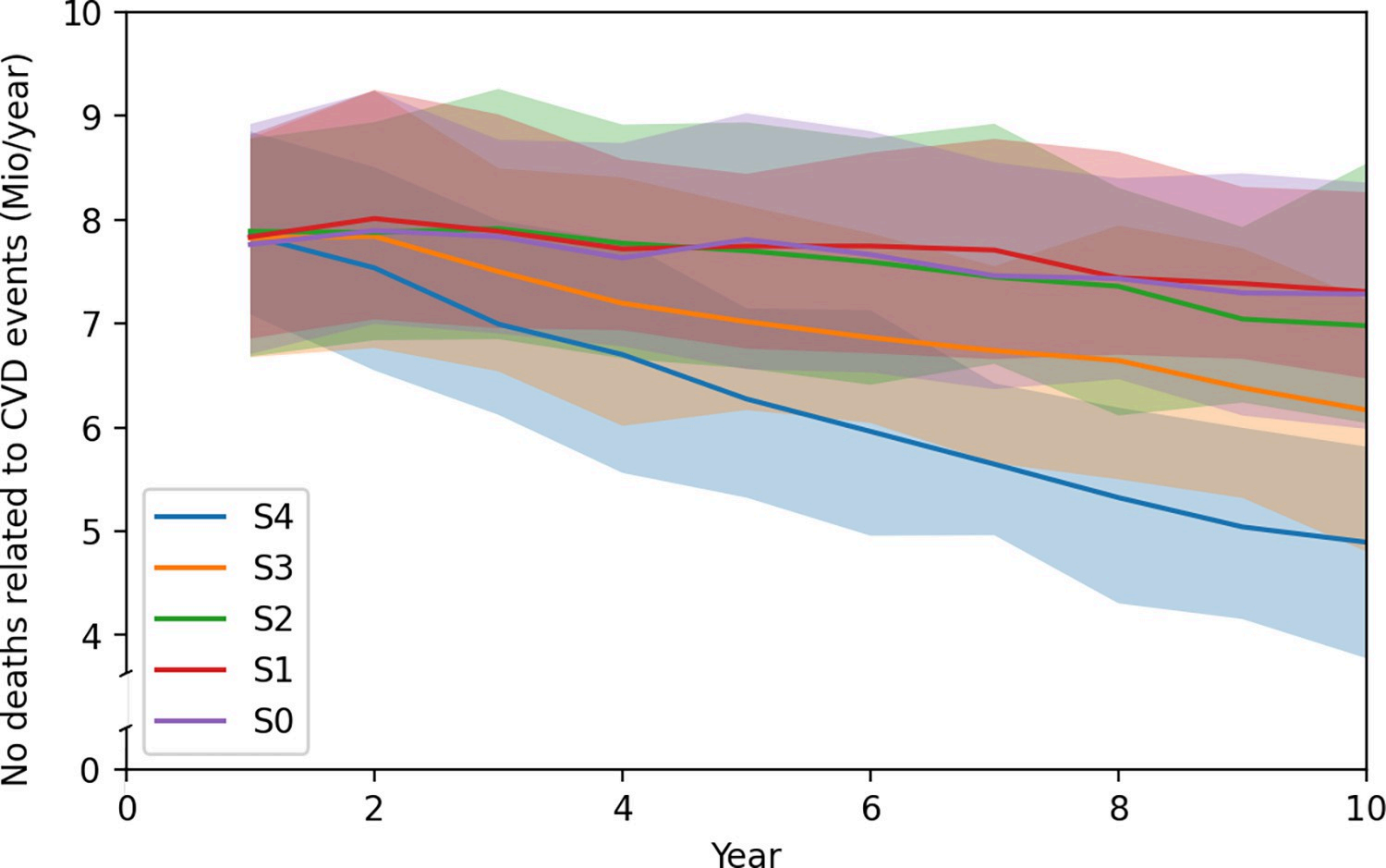
Mean numbers of deaths related to HTN-induced CVD events per year and their 95% credible intervals for the different scenarios for the population of LMICs (S0: Base case, S1: Lower accuracy, S2: Improved accuracy, S3: Increased coverage, S4: Increased coverage and screens completed). CVD, cardiovascular disease; HTN, hypertension; LMIC, low- and middle-income country.

Similar trends can be observed in the numbers of deaths resulting from a CVD event within the persons with HTN (Fig 4). Whereas survival does not improve much by increasing the test accuracy with 0.6% of HTN-induced deaths avoided over 10 years, we observe a high impact by doubling the coverage of the first screen (S3) with 6.6%. In year 10 alone, more than 2 million more lives (2,392,109) can be saved by additionally increasing completing probability in scenario S4, equalling to a total of 13,856,507 (13.7%) of the HTN-induced deaths over the 10 years.

When performing a subset analysis and comparing the differences of impact on the countries of same income, we observe similar patterns. Any diagnostic intervention has the most impact in UMICs, followed by LoMICs and LICs (See Fig B in S1 Text) due to the greater population with HTN and population size in these areas (Table 4).

## Discussion

This analysis provided insight on the potential effects of scaling up the screening for HTN for different scenarios. Across analyses, extending the coverage of a blood pressure measurement has a much higher potential impact than improving the accuracy of the test. Whereas the improvement of the test accuracy had only a small potential impact on CVD events and related mortality over 10 years (reduction of 0.5% and 0.6%, respectively), by doubling of the coverage of a first screen, 6.6% of HTN-induced deaths could be avoided over a period of 10 years, and by increasing screen completion in addition, even 13.7% of deaths would not occur.

The base case of our model was calibrated in such a way that the HTN prevalence and the increase in HTN prevalence over time is in line with the recent Global Burden of Disease (GBD) study report [3]. Moreover, according to a review on available data on HTN in the literature [30], in 2015, an estimated 7.5 million deaths were attributable to systolic blood pressure in LMICs. This number coincides with the number of deaths in our model for the baseline (S0). Additionally, in the baseline case (S0), the total number of people with HTN diagnosed per year over time and thus under control is decreasing (Fig 2B). This is in line with short-term trends in prevalence and control of HTN found in the literature [5,31,32].

These findings have implications on policy development and resource allocation. First of all, it shows that diagnosis and early identification plays a critical role in mitigating long-term mortality of CVD. Further, based on our data, programmatic expansion of screening interventions to scale up testing capacity might have a greater benefit than improving diagnostic accuracy. Models for scaling up testing could draw on successfully implemented community based mass screenings as implemented in Northern California [33] or annual HTN awareness campaigns, such as the May Measure Month [34]. This should be considered at the national and global level by funders of health interventions as well as in budget allocations for local healthcare improvements and international donor investments when weighing product versus access funding. Estimates of costs and cost effectiveness of the different scenarios are beyond the scope of this paper.

Although all efforts were made to mimic reality, our model also has several limitations. Due to the simplification of the population initialization, our model does not consider differences that arise from local heterogeneities. Moreover, genetic predisposition, ethnic background, family history of HTN, and dietary and exercise habits were not included in the model. Further, smoking and diabetes, which may contribute to HTN and CVD risk and their evolution over time were not included in the model due to lack of data on the direct relationship to parameterize the model. Furthermore, we acknowledge that blood pressure levels depend on more variables than what we can consider in the model, e.g., diet or lifestyle. The parametrization of the development of HTN was inferred from a logistic regression of the HPACC dataset and thus estimated prevalent and not incident hypertension. Also, we did not take into account adverse events accrued from putting false positively diagnosed individuals on treatment.

The model parameters (Table 2) are assumed to be valid across the entire population and main stratification factors. Furthermore, we adapted model input sources that stem from high-income countries (i.e., Framingham Heart Study) and scaled them to our purpose where possible. We assumed complete treatment adherence, which we acknowledge to be far from reality (i.e., full adherence to treatment is estimated to be only 10.3%) [9]. While this assumption is a simplification of the problems in the care cascade that follow the diagnosis, it enabled us to focus on the impact of diagnostics. The absolute reduction in the number of CVD-related deaths modeled would likely be lower in reality, though the relative reduction across our scenarios would still give realistic estimates given a likely similar treatment adherence. We further did not model chronic kidney disease as an outcome due to the limited data availability for the parametrization.

For the probability of being screened, we utilize numbers from Geldsetzer and colleagues [9] (at least one blood pressure measurement, diagnostic gap) to the population at large, even though they pertain only to persons with HTN. This might result in an overestimate in the absolute number of people screened, in particular when wanting to scale it down to a region or single country. In this case, the STEPS data can aid as additional external validation source [12]. Further, we do not consider the many years someone had HTN prior to a blood pressure measurement in detail. To reduce this bias, we assumed an estimate for the average years of opportunity for a blood pressure measurement and scaled it per annum. However, all model scenarios have the same underlying assumption and these parameters have little effect on the differences of the model scenarios, which is what we are primarily interested in.

Although the microsimulation added computational complexity compared to, for example, a model on a population level, it allowed us to integrate risk factor heterogeneity across individuals and include the possibility for each individual to develop CVD given their personal risk.

In conclusion, our simulation shows the promising impact in reducing HTN-induced morbidity and mortality in LMICs when increasing the coverage of testing for HTN and the improvement of screening completion; strategies to narrow the diagnostic gap in hypertension should focus on these two aspects.

## Supporting information

S1 STRESS Checklist STRESS checklist. (DOCX)

S1 Text Supporting information. (DOCX)

S1 HPACC Consortium Members of the HPACC Consortium. (DOCX)

## Abbreviations

BMI: body mass index
CVD: cardiovascular disease
GBD: Global Burden of Disease
HTN: hypertension
LIC: low-income country
LMIC: low- and middle-income country
LoMIC: lower-middle-income country
STEPS: STEPwise approach to noncommunicable diseases Surveillance
UMIC: upper-middle-income country
