# Supplementary material for: Addressing the diagnostic gap in hypertension through possible interventions and scale-up: A microsimulation study
Source: PLoS Med. 2022 Dec 6;19(12):e1004111. doi: 10.1371/journal.pmed.1004111 (PMC9725126; doi:10.1371/journal.pmed.1004111)
Supplement: S1 STRESS Checklist — (DOCX) [file pmed.1004111.s001.docx]

**Supplementary material to**

**Addressing the diagnostic gap in hypertension through possible interventions and scale up: a microsimulation study**

Lisa Koeppel, Sabine Dittrich, Sergio Brenner Miguel, Sergio Carmona, Stefano Ongarello, Beatrice Vetter, Jennifer Elizabeth Cohn, Till Baernighausen, Pascal Geldsetzer, Claudia M. Denkinger; HPACC Consortium

***S1: Strengthening the Reporting of Empirical Simulation Studies (STRESS)***

***System Dynamics guidelines STRESS-SD***

| **Section/Subsection** | **Item** | **Recommendation** | |
| --- | --- | --- | --- |
| 1. **Objectives** |  |  | |
| Purpose of the model | 1.1 | Introduction: par. 3 | |
| Model Outputs | 1.2 | CVD events (No and %), hypertension-induced deaths (No and %), prevented CVD events and deaths compared to baseline scenario S0 (No and %), number of newly diagnosed persons with hypertension, cumulative number of persons with hypertension diagnosed. | |
| 1. **Logic** |  |  | |
| Base model overview diagram | 2.1 | Methodology: Figure 1 | |
| Base model logic | 2.2 | Methodology: Simulation study design, par. 1 | |
| Scenario logic | 2.3 | Methodology: Simulation cycle, par. 1 - 4 | |
| Algorithms | 2.4 | Methodology, supplementary material S2 – S7 | |
| Components | 2.5 | 2.5.1 Stocks/Levels | Methodology: Population initialization, par. 1 - 3 |
|  |  | 2.5.2 Flows/Rates | Methodology: CVD risk and death probability calculations par. 1 - 3, Table 2, supplementary material S2 – S7 |
|  |  | 2.5.3 Constants / Converters / auxiliary variables | Table 1, Table 2, supplementary material S2 – S7 |
|  |  | 2.5.4 Graphical functions/lookup tables | / |
|  |  | 2.5.5 Sources and Sinks | Discussion: par. 4 – 6 |
| 1. **Data** |  |  | |
| Data sources | 3.1 | Table 2, Global burden of disease data | |
| Pre-processing | 3.2 | Before bootstrapping from the Global burden of hypertension dataset, we limited it to people aged 30 and older. | |
| Input parameters | 3.3 | Supplementary material S2 – S7, Table 2 | |
| Assumptions | 3.4 | Methodology section. Full section | |
| 1. **Experimentation** |  |  | |
| Initialisation | 4.1 | Supplementary material S2 – S7, Table 2 | |
| Run length | 4.2 | Microsimulation per year with a burn-in phase of 3 years and an observation period of 10 years. | |
| Estimation approach | 4.3 | / | |
| 1. **Implementation** |  |  | |
| Software or programming language | 5.1 | Python 3.9.2  NumPy 1.20.1  Pandas 1.2.5  Matlotlib 3.3.4  SciPy 1.6.1  Swifter 1.0.9  Scikit-learn 0.24.2 | |
| Random sampling | 5.2 | NumPy library numpy.random | |
| Model execution | 5.3 | / | |
| System Specification | 5.4 | Model runtime: 35 minute for one simulation, parallelized on a **High End Computing (HEC) Cluster:** The combined facility offers 10,000 cores, 49TB of aggregate memory, 24 Tesla V100 GPUs, 230TB of high performance filestore for general use and 4PB of medium performance filestore for GridPP data. | |
| 1. **Code Access** |  |  | |
| Computer Model Sharing Statement | 6.1 | The source code is available under:  https://zenodo.org/record/6796368 | |
