# Supplementary material for: Addressing the diagnostic gap in hypertension through possible interventions and scale-up: A microsimulation study
Source: PLoS Med. 2022 Dec 6;19(12):e1004111. doi: 10.1371/journal.pmed.1004111 (PMC9725126; doi:10.1371/journal.pmed.1004111)
Supplement: S1 Text — (DOCX) [file pmed.1004111.s002.docx]

**Supplementary material to**

**Addressing the diagnostic gap in hypertension through possible interventions and scale up: a microsimulation study**

Lisa Koeppel, Sabine Dittrich, Sergio Brenner Miguel, Sergio Carmona, Stefano Ongarello, Beatrice Vetter, Jennifer Elizabeth Cohn, Till Baernighausen, Pascal Geldsetzer, Claudia M. Denkinger; HPACC Consortium

***Section A: Population size***

In order to deal with the complexity of evaluating this microsimulation model, we performed the calculations on the respective subsets and scaled the outcomes to the fit the desired population size of 3.15 billion (LIC: 167,113,750; LoMIC: 1,360,371,000; UMIC: 1,621,405,000). This was determined as follows:

The minimum age in our simulation was 30 years to account for the fact that hypertension is less prevalent in the younger generation. As the age distribution is different in the respective country income classes, we chose the population proportion of individuals of 30 years and older according to the age pyramids from Uganda (25% of total country population) and India (46.7%) as representatives for LIC and LoMIC, respectively [1, 2]. For UMIC, we chose the age distribution representative for the populations of China (63%) and Peru (50.5%) [3, 4]. Out of the total population of 6.44 billion people living in LMIC, the population aged 30 years an older resulted in around 3.15 billion (LIC: 167,113,750; LoMIC: 1,360,371,000; UMIC: 1,621,405,000) [5]. We did not model any births into the population over time because we assumed the period of ten years to be too short to result in major demographic changes.

***Section B: Assignment of mortality dates***

We chose a Weibull distribution for modelling the mortality dates *X* for the individuals.

We obtained the mean mortality age *E(X)* from the literature ([6]) and were thus able to reparametrize the scale parameter λ by means of the shape parameter κ:

$\lambda=E\left( X \right)/{Gam}\left( 1+1/\kappa\right)$, with Gam() denoting the Gamma function. In this way, we obtained a single parameter distribution depending only on κ. The shape parameter κ was then empirically adapted such that the probability of surviving until a certain age was adapted to the data of the age pyramid of the representative countries for the income groups ([1-4]). In detail, we chose κ to be 12 for LICs, and 5 for LMICs and UMICs respectively.

***Section C: CVD risk function definition***

We adapted the sex-specific Cox proportional hazard model from [7] and altered it to our needs. The probability of experiencing a CVD event within one year for individual *j* is given by

$$p_{j}=\left[ 1-S_{0}\left( 1 \right)^{exp\left[ \left( \sum\beta_{i}\left( X_{i}-\bar{X_{i}} \right) \right)+r_{j} \right]} \right]{*c_{j}*0.8}^{m_{j}}$$

where

- $S_{0}\left( 1 \right)$is the baseline survival after one year.

In [7] we are given the sex-specific baseline hazard for $S_{0}\left( 10 \right)$(0.88431 for men and 0.94833 for women). Assuming that the baseline hazard rate is constant over time, then $S_{0}\left( t \right)=exp\left( -\lambda t \right)$ with $\lambda=-ln\left( S_{0}\left( 10 \right) \right)/{10}$

Other baseline survival functions were analysed as well, not providing substantially different results due to the close proximity to 1.

- $\beta_{i}$is the estimated log hazard ratio for covariate *i* (age: women 2.72107, men 3.11296; BMI: women 0.51125, men 0.79277; systolic blood pressure if treated: women 2.88267, men 1.92672, systolic blood pressure if not treated: women 2.81291, men 1.85508).
- $X_{i}-\bar{X_{i}}$is the difference from the mean value of the i-th log-transformed covariate.
- $r_{j}$ is the additional risk of an individual *j* after having experienced a CVD event before. We interpolated the risk for a recurrent CVD event with an exponential function r(t) = 2.46813797 exp(-0.03383494*t) with *t* denoting the years after the last CVD event. This results in a hazard ratio of 2.4 at year 1, hazard ratio of 2.2 at year 3 and hazard ratio of 2.1 at year 5 [8]. If no CVD event was experienced before, there is no additional risk, i.e. $r\left( 0 \right):=0$.
- $c_{j}$ is the factor to scale the risk to LMICs.
- $m_{j}$ is the reduction of systolic blood pressure due to treatment adherence in 10 mmHg.

The probability $a_{c}$ which specific CVD event *c* takes place (MI, stroke or other CVD) is calculated by the weighted probabilities

$$a_{c}=\frac{d_{c}{HR}_{c}}{\sum d_{k}{HR}_{K}}$$

- - $d_{k}$stands for the proportion of specific event among all events (MI 46.7%, stroke 35.5%, other CVD 17.8% [9])
  - *HR* is the weight or additional risk (hazard ratio) associated with a past event compared to the normal population. The specific values are provided in Table 2 in the manuscript. If an individual has not experienced an event *k* yet, then ${HR}_{k}=1$.
  - Hazard of recurrence of stroke was fitted with the exponential function:

F(x) = 17.043290497012492 * exp(-0.127706405 * x)

- - Hazard of recurrence of MI was fitted with the exponential function:

F(x) = 3.157925925 * exp(-0.0391659216 * x)

***Section D: Completion of screen calculation***

From the subgroup of first screened individuals, a proportion (see Table 3) was enabled to complete their screen and get diagnosed. The people with a higher CVD risk were more likely to complete their screen. In specific, we ranked the population with respect to their CVD risk. The probability of completing the screen for each individual was then calculated by its proportion on the ranking multiplied by the completion probability. For example, assume 4 individuals were screened once. The person with the highest CVD risk (rank 4) had the probability of 4/4 *CompletionProbability of completing the screen, whereas the person with the second highest risk (rank 3) had probability 3/4 *CompletionProbability. By comparing to a sample from a U(0,1) distribution we then evaluated the outcome of a second screen.

***Section E: Hypertension development prediction model***

The logistic regression model was trained on the HPACC dataset with the following estimates:

$\beta_{intercept}=-5.50261742$, $\beta_{age}=0.05872852$, $\beta_{bmi}=0.08370991$,

$$\beta_{sex}=-0.17200178$$

This describes the probability for getting hypertension once throughout the whole simulation. Thus, we scale it down to one year of the observation period by dividing the estimated probability by the number of observation years, that is $\frac{1}{10}\frac{exp(\sum\beta_{i}x_{i})}{exp(\sum\beta_{i}x_{i})+1}$ .

***Section F: White-coat / Masked Hypertension***

About 15% to 30% people with an elevated office-blood pressure have white-coat hypertension defined as temporarily elevated blood pressure from before to during the visit in a clinical setting [10]. White-coat hypertensive persons do not experience a greater risk for CVD events than the normotensive control population [10]. Thus, in our model we treated white-coat hypertensive people as persons with a normal blood pressure for the calculation of the CVD risk. The contrary phenomenon, masked hypertension, describes a normal blood pressure in the clinical setting and elevated otherwise. Due to the higher CVD risk associated with masked hypertension, it was modelled with a high systolic blood pressure.

**Table A: Differences in proportions of the HPACC dataset [11]**

| **Parameter** | **HPACC**  **dataset** | **HPACC** **dataset, age >= 30 years** |
| --- | --- | --- |
| Raised systolic blood pressure  (>= 140 mmHg) prevalence  LIC LOMIC  UMIC | 26.0%  16.1%  30.7% | 30.6% 24.2% 37.5% |
| Overweight (BMI >= 25)  LIC  LOMIC  UMIC | 27.9% 24.3%  62.6% | 29.8% 34.1% 68.7% |
| Sex distribution (% male)  LIC  LOMIC  UMIC | 40.0% 18.6%  42.0% | 41.4% 20.9% 41.4% |

Fig A: Heatmaps of scenarios compared to baseline scenario S0.

1. % Prevented CVD events in people with HTN / year


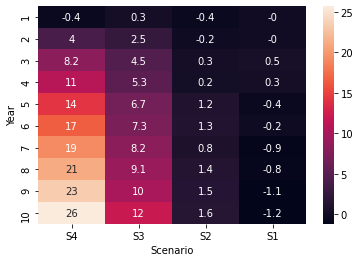


1. % Prevented deaths related to CVD events / year


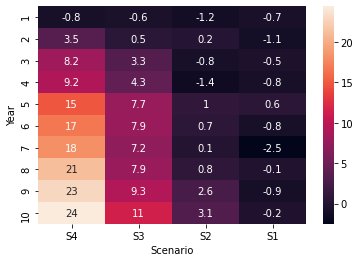


1. % New persons with HTN diagnosed / year


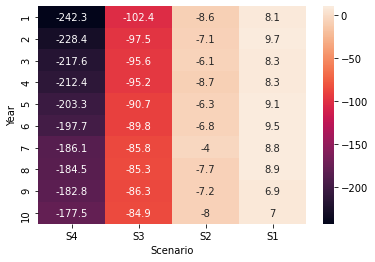


1. % Total No persons with HTN diagnosed


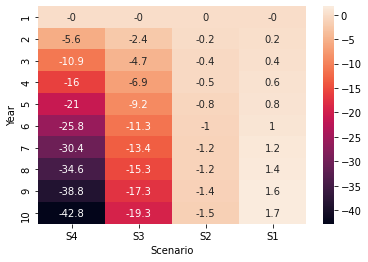


Fig B: Stratification by income class.

1. Low-Income countries


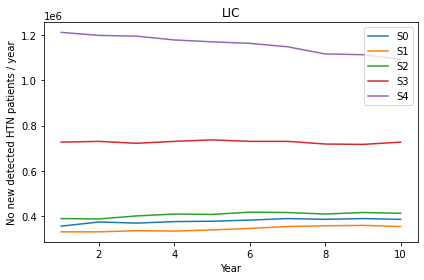


1. Lower-Middle Income countries


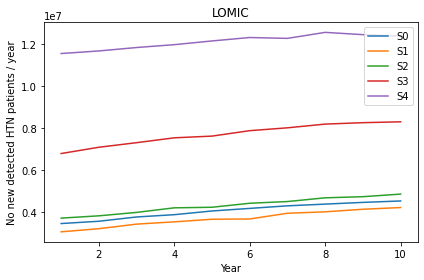


***
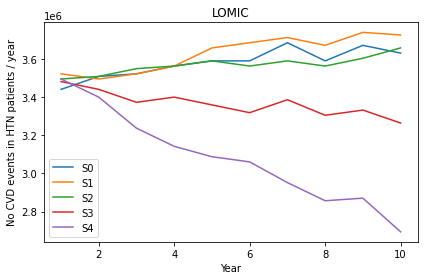
***

1. Upper-Middle-Income countries


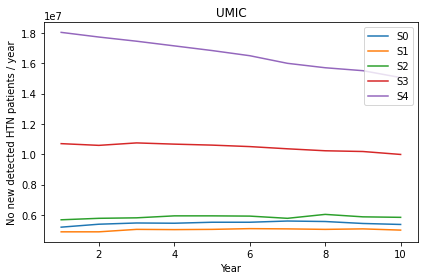


***
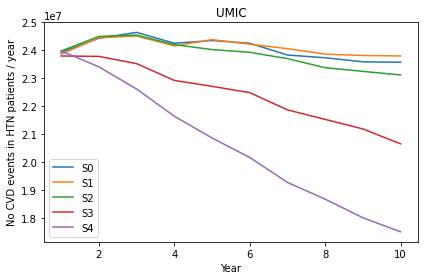
***

**References**

1. Pyramid, P. *Population India 2019*. [cited 2021 Nov, 5th]; Available from: <https://www.populationpyramid.net/india/2019/>.

2. Pyramid, P. *Population Uganda 2019*. [cited 2021 Nov, 5th]; Available from: <https://www.populationpyramid.net/uganda/2019/>.

3. Pyramid, P. *Population China 2019*. [cited 2021 Nov, 5th]; Available from: <https://www.populationpyramid.net/china/2019/>.

4. Pyramid, P. *Population Peru 2019*. [cited 2021 Nov, 5th]; Available from: <https://www.populationpyramid.net/peru/2019/>.

5. Group, T.W.B. *Population, total*. 2021 [cited 2021 Nov, 5th]; Available from: <https://data.worldbank.org/indicator/SP.POP.TOTL>.

6. Group, T.W.B. *Life expectancy at birth, total (years)*. 2021 [cited 2021 Nov, 5th]; Available from: <https://data.worldbank.org/indicator/SP.DYN.LE00.IN>.

7. D'Agostino, R.B., Sr., et al., *General cardiovascular risk profile for use in primary care: the Framingham Heart Study.* Circulation, 2008. **117**(6): p. 743-53.

8. Edwards, J.D., et al., *Long-term morbidity and mortality in patients without early complications after stroke or transient ischemic attack.* Cmaj, 2017. **189**(29): p. E954-e961.

9. Bowry, A.D., et al., *The Burden of Cardiovascular Disease in Low- and Middle-Income Countries: Epidemiology and Management.* Can J Cardiol, 2015. **31**(9): p. 1151-9.

10. Franklin, S.S., et al., *White-coat hypertension: new insights from recent studies.* Hypertension, 2013. **62**(6): p. 982-7.

11. Manne-Goehler, J., et al.,. *Data Resource Profile: The Global Health and Population Project on Access to Care for Cardiometabolic Diseases (HPACC)*. Int J Epidemiol, 2022.

**List of Tables**

[Table A: Differences in proportions of the HPACC dataset [11] 4](#_Toc115246006)

**List of Figures**

[Fig A: Heatmaps of scenarios compared to baseline scenario S0. 5](#_Toc108400806)

[Fig B: Stratification by income class. 7](#_Toc108400807)
