# Supplementary material for: Addressing the diagnostic gap in hypertension through possible interventions and scale-up: A microsimulation study
Source: PLoS Med. 2022 Dec 6;19(12):e1004111. doi: 10.1371/journal.pmed.1004111 (PMC9725126; doi:10.1371/journal.pmed.1004111)
Supplement: S1 HPACC Consortium — (DOCX) [file pmed.1004111.s003.docx]

**Supplementary material to**

**Addressing the diagnostic gap in hypertension through possible interventions and scale up: a microsimulation study**

Lisa Koeppel, Sabine Dittrich, Sergio Brenner Miguel, Sergio Carmona, Stefano Ongarello, Beatrice Vetter, Jennifer Elizabeth Cohn, Till Baernighausen, Pascal Geldsetzer, Claudia M. Denkinger; HPACC Consortium

**HPACC Consortium members**

| Rifat Atun | Department of Global Health and Population, Harvard T. H. Chan School of Public Health, Boston, United States of America  Department of Global Health and Social Medicine, Harvard Medical School, Harvard University, Boston, United States of America |
| --- | --- |
| Glennis Andall-Brereton | Private Public Health Consultant, Port of Spain, Trinidad and Tobago |
| Silver Bahendeka | Department of Internal Medicine, St. Francis Hospital \| MKPGMS Uganda Martyrs University, Kampala, Uganda |
| Brice Bicaba | Institut National de Santé Publique, Burkina Faso |
| Norov Bolormaa | Nutrition division, National Center for Public Health, Ulaanbaatar, Mongolia |
| Garry Brian | The Fred Hollows Foundation New Zealand |
| Farshad Farzadfar | Non-Communicable Diseases Research Center, Endocrinology and Metabolism Population Sciences Institute, Tehran University of Medical Sciences, Tehran, Iran |
| Yosef Farzi | Non-Communicable Diseases Research Center, Endocrinology and Metabolism Population Sciences Institute, Tehran University of Medical Sciences, Tehran, Iran |
| David Guwatudde | Department of Epidemiology and Biostatistics,  School of Public Health,  Makerere University,  Kampala, Uganda |
| Krishna Kumar Aryal | The Bergen Center for Ethics and Priority Setting, Department of Global Public Health and Primary Care, Faculty of Medicine, University of Bergen, Bergen, Norway |
| Demetre Labadarios | Professor Emeritus, Faculty of Medicine and Health Sciences, Stellenbosch University, South Africa |
| Nuno Lunet | Departamento de Ciências da Saúde Pública e Forenses e Educação Médica, Faculdade de Medicina, Universidade do Porto, 4200-319 Porto, Portugal.  EPIUnit, Instituto de Saúde Pública da Universidade do Porto, 4050-600 Porto, Portugal.  Laboratório para a Investigação Integrativa e Translacional em Saúde Populacional (ITR), 4050-600 Porto, Portugal. |
| Joao Martins | Faculty of Medicine and Health Sciences, Universidade Nacional Timor Lorosa'e, Dili, Timor-Leste |
| Masoud Masinaei | Non-Communicable Diseases Research Center, Endocrinology and Metabolism Population Sciences Institute, Tehran University of Medical Sciences, Tehran, Iran |
| Sahar Saeedi Moghaddam | Endocrinology and Metabolism Research Center, Endocrinology and Metabolism Clinical Sciences Institute, Tehran University of Medical Sciences, Tehran, Iran  Kiel Institute for the World Economy, Kiel, Germany |
| Sarah Quesnel-Crooks | Chronic Diseases and Injuries Department, Caribbean Public Health Agency (CARPHA), Port of Spain, Trinidad and Tobago |
| Abla M. Sibai | Epidemiology and Population Health Department, Faculty of Health Sciences American University of Beirut, Beirut, Lebanon |
| Michaele Theilmann | Heidelberg Institute of Global Health (HIGH), Medical Faculty and University Hospital, University of Heidelberg, Heidelberg, Germany. |
| Sebastian Vollmer | Department of Economics & Centre for Modern Indian Studies, University of Goettingen, Germany |
| Zhaxybay Zhumadilov | Nazarbayev University School of Medicine, Astana city, Kazakhstan |
